# How have the demographic and practice patterns of older general practitioners in rural and remote Australia changed over the past decade?

**DOI:** 10.1186/s12913-026-15090-0

**Published:** 2026-07-30

**Authors:** Saliu Balogun, Chipampe Masongo, Hafis Tijani, Fiona Doolan-Noble

**Affiliations:** 1 Goldfields University Department of Rural Health, Curtin University, Kalgoorlie Campus,, Kalgoorlie, Western Australia Australia; 2 Mernda GP Clinic, Mernda, VIC Australia

**Keywords:** General practitioners, Rural health, Workforce demographics, Ageing workforce

## Abstract

**Background:**

Late-career general practitioners (GPs) aged 65 and over play a vital role in rural health care but have been underrepresented in research and policy discussions, despite increasing concerns about workforce ageing in Australia. This study aimed to describe changes in the demographics and work settings of late-career GPs in rural and regional Australia over the past decade, and to compare these trends with metropolitan areas.

**Methods:**

Data from the National Health Workforce Dataset from 2013 to 2024 were analysed. Analyses were restricted to GPs aged 65 years and older who were registered and practising in Australia. GP practice locations were classified as metropolitan (MMM1), regional (MMM2), or rural and remote (MMM3–7) using the Modified Monash Model (MMM). Descriptive statistics and trend analyses were used to identify changes in demographics and work settings over the decade.

**Results:**

The number of late-career GPs increased across all regions between 2013 and 2024: by 82.6% in rural and remote areas (454 to 829), 67.5% in regional areas (209 to 350), and 67.5% in metropolitan areas (2,523 to 4,226). Female representation increased over time, particularly in rural (15.2% to 26.9%) and metropolitan areas (19.9% to 33.1%), but remained low in regional areas (1.7% to 3.3%). There was an increase in the proportion of Australia/New Zealand-trained GPs across all regions: rural (70.4% to 72.8%), regional (57.8% to 68.6%), and metropolitan (60.0% to 61.2%). However, Indigenous representation remained low across all regions throughout the study period. Rural late-career GPs predominantly worked in private settings. The number working in hospital settings and Aboriginal health organisations increased over time. The proportion of late-career GPs intending to retire within five years increased across all regions: rural (50.2% to 64.5%), regional (56.3% to 67.5%), and metropolitan areas (40.4% to 53.6%).

**Conclusions:**

The late-career GP workforce in Australia is growing, becoming more gender-diverse, and increasingly locally trained. However, it continues to face challenges with Indigenous representation and impending retirements, particularly in already underserved rural and remote areas.

**Supplementary Information:**

The online version contains supplementary material available at https://doi.org/10.1186/s12913-026-15090-0.

## Introduction

The general practitioner (GP) workforce is critical to the delivery of primary healthcare in Australia [1]. Over recent decades, the demographic profile of the Australian GP workforce has shifted, with an increase in the proportion of older GPs [2]. The trend toward an older workforce presents both opportunities and potential challenges. On the one hand, late-career GPs (aged 65 years and older) contribute irreplaceable clinical experience that can only be obtained through years of practice, and they also provide mentorship for younger practitioners [3, 4]. Older GPs are frequently embedded in their communities, possess deep knowledge of local health needs, and provide continuity of care, particularly in rural and remote areas that are vulnerable to workforce shortages and high turnover [3, 5]. Many late-career GPs continue to practise well beyond the conventional retirement age, driven by professional commitment, community needs and personal preference [4, 6]. On the other hand, the growing proportion of late-career GPs raises concerns about the sustainability of the primary care workforce, as older GPs approach retirement and the pipeline of new practitioners remains challenging [7, 8]. These challenges are particularly critical in rural and remote areas that are characterised by persistent recruitment and retention difficulties [3, 7].

Despite their critical roles in primary care, late-career GPs have received limited attention in rural health workforce research. Most workforce analyses focus on the recruitment and retention of early to mid-career GPs [9–11]. To develop strategies to sustain rural primary care services in an environment characterised by an ageing health workforce, it is important to understand changes in the demographics and practice patterns of late-career GPs. Therefore, this study aims to investigate (1) how the demographic and professional profiles of late-career GPs in rural and remote areas of Australia have changed over the past decade, and (2) how changes in the profile of rural late-career GPs compare with those in metropolitan areas.

## Data and methods

### Data source and ethical considerations

Data from the annual National Health Workforce Dataset (NHWDS) from 2013 to 2024 were analysed. Medical practitioners are regulated by the Australian Health Practitioner Regulation Agency (AHPRA), and these professionals are required to renew their registration annually to maintain their practice license [12]. The NHWDS comprises data collected during AHPRA’s annual registration process and from a voluntary workforce survey that is also completed during the registration process [12]. The dataset has an excellent response rate, with a 2023 response rate of 97.2% for medical practitioners [12]. The dataset is managed by the Australian Government Department of Health and Aged Care, which took over custodianship from the Australian Institute of Health and Welfare (AIHW) in July 2016.

### Participants and study location

GPs registered with AHPRA and working in their registered profession in Australia from 2013 to 2024 were included in the study. Practitioners registered with AHPRA but practising internationally were excluded. The GPs’ location of practice was categorised as metropolitan, regional, or rural according to the 2019 Monash Modified Model (MMM) [13]. The MMM is a geographically based classification system developed to better reflect access to services across metropolitan, regional, rural, and remote areas of Australia [13]. The MMM assigns every location in Australia to one of seven categories (MMM 1–7) based on population size and remoteness. MMM 1 refers to metropolitan areas, comprising major cities. MMM 2 comprises regional centres, defined as locations in, or within 20 km by road of a town with a population greater than 50,000. MMM 3 are large rural towns that are in, or within 15 km by road of a town with a population between 15,000 and 50,000. MMM 4 includes medium rural towns located in, or within 10 km by road of a town with a population between 5,000 and 15,000. MMM 5 are small rural towns. MMM 6 and MMM 7are classified as remote or very remote communities, respectively [13]. The term ‘rural’ is used in this study to collectively refer to MMM3–7, which includes rural, remote, and very remote locations [13]. The term ‘rural’ is used in this study to collectively refer to MMM3–7, which includes rural, remote, and very remote locations. The MMM classification was modified in 2023, and the NHWD provides both the 2019 and 2023 MMM classifications. This study uses the 2019 classification, and there are no changes in the number of late-career GPs classified as working in rural areas as a result of changes in the MMM classification in 2023 (Supplementary Table 1).

### Measures and data analysis

Demographic and employment data were obtained from the National Health Workforce Data Tool. Variables examined included sex (male vs. female), Indigenous status (Indigenous vs. non-Indigenous), initial country of registration (Australia/New Zealand vs. overseas), years in practice (≤ 10 years, > 10 years), intended years remaining in practice (< 5 years, 5–10 years, > 10 years), total weekly hours worked (1–19, 20–34, 35–49, ≥ 50 h), and work setting. Descriptive statistics were used to summarise workforce characteristics over time. Counts and proportions were calculated for categorical variables, and trends from 2013 to 2024 were visualised using line graphs stratified by region. The National Health Workforce Data Tool applies perturbation to counts ranging from 0 to 3 to maintain confidentiality [14]. These values are reported as < 5, and percentages were not reported for variables with perturbed cell counts. Temporal trends and differences across regions were estimated using binomial generalised linear models with a logit link function. The year variable was modelled as a continuous variable (centred at 2013) to estimate average annual changes. Interaction terms between year and geographic region were included to assess whether trends differed across metropolitan, regional, and rural areas. Results are presented as average marginal effects (AME), interpreted as percentage-point (pp) change per year. All analyses were conducted using Stata version 19 (StataCorp, College Station, TX, USA).

## Results

The absolute number of late-career GPs registered and practising in Australia increased across all regions between 2013 and 2024 (Table 1). In rural areas, the number of GPs increased from 454 in 2013 to 829 in 2024; in regional areas, from 209 to 350; and in metropolitan areas, from 2523 to 4226. As a proportion of the total GP workforce in each region, late-career GPs increased from 12.4% to 14.6% in rural and remote areas, from 15.5% to 17.3% in metropolitan areas, but remained relatively stable in regional areas (2013: 11.5% vs. 2024: 11.8%).

**Table 1:** Profile of late-career general practitioners registered and practising their professions in metropolitan (MMM 1), regional (MMM2) and rural and remote (MMM 3–7) areas of Australia

	Rural and Remote (*n*, %)		Regional (*n*, %)		Metropolitan (*n*, %)	
	2013	2024	2013	2024	2013	2024
Number of practitioners	454 (12.4%)†	829 (14.6%)†	209 (11.5%)†	350 (11.8%)†	2523 (15.5%)†	4226 (17.3%)†
**Sex**						
Male	385 (84.8%)	606 (73.1%)	2022 (98.3%)	2829 (96.7%)	2022 (80.1%)	2829 (66.9%)
Female	69 (15.2%)	223 (26.9%)	43 (1.7%)	96 (3.3%)	501 (19.9%)	1397 (33.1%)
**Indigenous status**						
Indigenous	< 5*	< 5*	< 5*	< 5*	5 (0.2%)	19 (0.4%)
Not Indigenous	> 445*	> 820*	> 200*	> 340*	2518 (99.8%)	4207 (99.6%)
**Initial Country of registration**						
Australia/NZ	311 (70.4%)	602 (72.8%)	118 (57.8%)	240 (68.6%)	1449 (60.0%)	2578 (61.2%)
Other overseas	131 (29.6%)	225 (27.2%)	86 (42.2%)	110 (31.4%)	967 (40.0%)	1637 (38.8%)
**Years in practice**						
Ten years or less	5 (1.2%)	11 (1.3%)	8 (3.9%)	9 (2.6%)	26 (1.0%)	77 (1.8%)
More than 10 years	425 (98.8%)	810 (98.7%)	196 (96.1%)	341 (97.4%)	2316 (98.9%)	4119 (98.2%)
**Years intended to work**						
Less than 5 years	208 (50.2%)	527 (64.5%)	107 (56.3%)	235 (67.5%)	922 (40.4%)	2235 (53.6%)
Five to 10 years	180 (43.5%)	268 (32.8%)	71 (37.4%)	101 (29.0%)	1208 (53.0%)	1767 (42.3%)
More than 10 years	26 (6.3%)	22 (2.7%)	12 (6.3%)	12 (3.4%)	150 (6.6%)	171 (4.1%)
**Total hours worked per week**						
1–19 h	68 (15.0%)	128 (15.4%)	39 (18.5%)	61 (17.5%)	388 (15.4%)	763 (18.1%)
20–34 h	119 (26.2%)	238 (28.7%)	50 (23.7%)	146 (28.7%)	781 (31.0%)	1545 (36.6%)
35–49 h	150 (33.0%)	314 (37.9%)	75 (35.5%)	103 (29.6%)	890 (35.3%)	1382 (32.7%)
50 h or more	117 (25.8%)	149 (18.0%)	45 (17.5%)	40 (11.4%)	464 (18.4%)	536 (12.7%)
**Work settings**						
Private practice	393	613 (73.9%)	184	291 (83.1%)	2352 (93.2%)	3841 (90.9%)
Aboriginal health service	19	83 (10.0%)	< 5*	11 (3.1%)	6 (0.2%)	22 (0.5%)
Hospital	< 5*	61 (7.3%)	< 5*	17 (4.9%)	28 (1.1%)	68 (1.6%)
Other work settings**	> 30*	73 (8.8%)	> 10*	31 (8.9%)	137 (5.4%)	295 (7.0%)

*Values between 0 and 3 were perturbed in the original dataset to preserve confidentiality and have been reported as < 5. Percentages were not calculated for affected cells or columns

†: Late-career GP as a percentage of the total GP workforce for each region

**Other work settings include residential facilities, community health care services, correctional services, and the defence forces/other government agencies

**Fig. 1:**
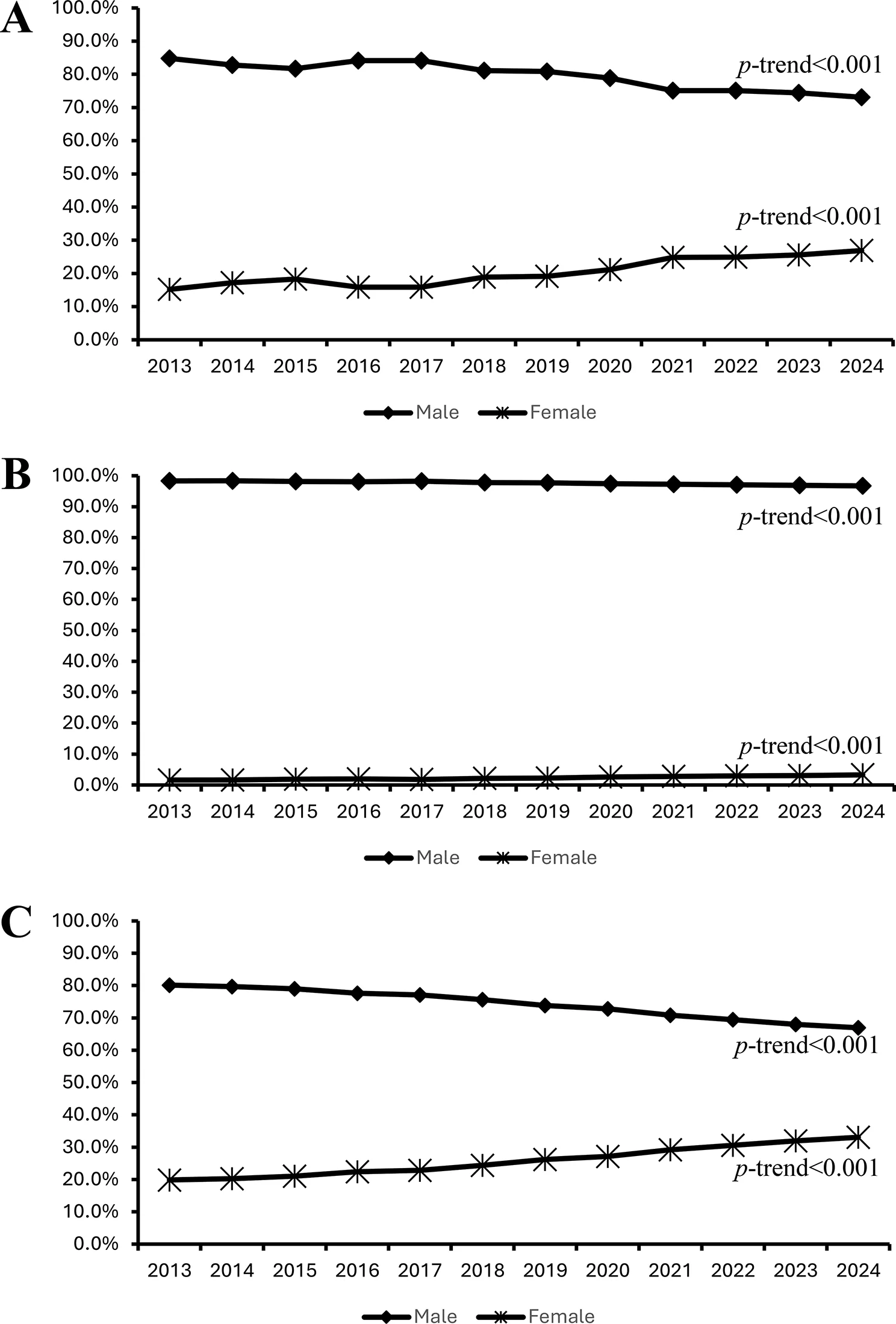
Changes in gender of late-career General Practitioners in rural/remote (**A**), regional (**B**) and metropolitan (**C**) areas in Australia from 2013 to 2024

There is a linear, increasing trend in the proportion of late-career female GPs in rural areas, from 15.2% in 2013 to 26.9% in 2024 (p-trend < 0.001; Fig. 1A). This is accompanied by a decrease in the proportion of male late-career GPs (84.8% in 2013 vs. 73.1% in 2024; p-trend < 0.001). Nevertheless, the proportion of male late-career GPs remained higher than female throughout the study period. This pattern is also observed in metropolitan areas, where women accounted for 33.1% of late-career GPs in 2024, up from 19.9% in 2013 (Fig. 1C). Regional areas have the lowest proportion of female late-career GPs relative to males (Fig. 1B), and the region had the smallest change in the gender composition over the study period. For instance, the average annual percentage points increase in female late-career GPs in regional areas was 1.05 (95% CI: 0.95–1.14, *p* < 0.001) compared to 1.09 (95% CI: 0.91–1.27, *p* < 0.001) in rural areas and 1.28 (95% CI: 1.21–1.35, *p* < 0.001) in metropolitan areas (Table 2). The absolute number of late-career Indigenous practitioners in rural and regional areas was low throughout the study period. Only 0.2% and 0.4% of late-career GPs in metropolitan areas were identified as Indigenous in 2013 and 2024, respectively. There was a very small but statistically significant increase in the proportion of Indigenous late-career GPs across all regions (rural: 0.05 pp per year, 95% CI: 0.03–0.07; *p* < 0.001; regional areas: 0.15 pp per year; 95% CI: 0.06–0.23; *p* = 0.001; metropolitan areas 0.01 pp per year; 95% CI: 0.00–0.03; *p* = 0.035).

**Table 2:** Estimated average annual change (from 2013 to 2024) in the proportion of late career general practitioners’ characteristics and practice patterns, stratified by geographical region

Variables	Rural/Remote		Regional		Metropolitan	
	AME (95% CI)	*p*–values	AME (95% CI)	*p*–values	AME (95% CI)	*p*–values
**Sex**						
Female (vs. male)	1.09 (0.91, 1.27)	< 0.001	1.05 (0.95, 1.14)	< 0.001	1.28 (1.21, 1.35)	< 0.001
**Indigenous status**						
Indigenous (vs. non-Indigenous)	0.05 (0.03, 0.07)	< 0.001	0.15 (0.06, 0.23)	0.001	0.01 (0.00, 0.03)	0.035
**Initial Country of registration**						
Other overseas (vs. Australia/New Zealand)	–0.45 (–0.65, − 0.24)	< 0.001	–1.45 (–2.00, − 0.90)	< 0.001	–0.18 (–0.43, 0.06)	0.147
**Years in practice**						
Less than 10 years (vs ten years or more)	–0.07 (–0.14, − 0.003)	0.041	–0.07 (–0.17, 0.04)	0.224	0.06 (0.01, 0.10)	0.020
**Years intended to work**						
More than 10 years (vs less than 5 years)	–1.35 (–1.60, − 1.10)	< 0.001	–1.60 (–2.21, − 0.98)	< 0.001	–1.20 (–1.31, − 1.08)	< 0.001
Five to 10 years (vs less than 5 years)	–0.46 (–0.69, − 0.23)	< 0.001	–0.41 (–0.67, − 0.15)	0.002	–0.63 (–0.72, − 0.55)	< 0.001
**Total hours worked per week**						
20–34 h (vs 1-19h)	0.23 (–0.07, 0.53)	0.127	0.65 (0.14, 1.16)	0.012	–0.06 (–0.26, 0.14)	0.554
35–49 h (vs 1-19 h)	0.29 (0.08, 0.50)	0.007	–0.48 (–0.95, − 0.01)	0.046	–0.54 (–0.73, − 0.35)	< 0.001
50 h or more (vs 1-19h)	–4.50 (–5.77, − 3.22)	< 0.001	–6.06 (–8.68, − 3.44)	< 0.001	–4.99 (–5.91, − 4.07)	< 0.001
**Work settings**						
Aboriginal health service (vs private practice)	0.58 (0.48, 0.68)	< 0.001	0.17 (0.03, 0.31)	0.015	0.03 (0.02, 0.04)	< 0.001
Hospital (vs private practice)	0.39 (0.26, 0.51)	< 0.001	0.20 (0.17, 0.23)	< 0.001	–0.03 (–0.03, − 0.02)	< 0.001
Other work settings** (vs private practice)	0.22 (–0.09, 0.53)	0.171	0.26 (0.01, 0.51)	0.045	0.26 (0.22, 0.30)	< 0.001

AME: Average marginal effects, representing the estimated average annual change in the predicted proportion (percentage points per year) from 2013 to 2024. For each comparison, the category listed first was modelled relative to the reference category shown in parentheses. Positive AMEs indicate an increasing proportion of the first category over time, whereas negative AMEs indicate a decreasing proportion. **Other work settings include residential facilities, community health care services, correctional services, and the defence forces/other government agencies

**Fig. 2:**
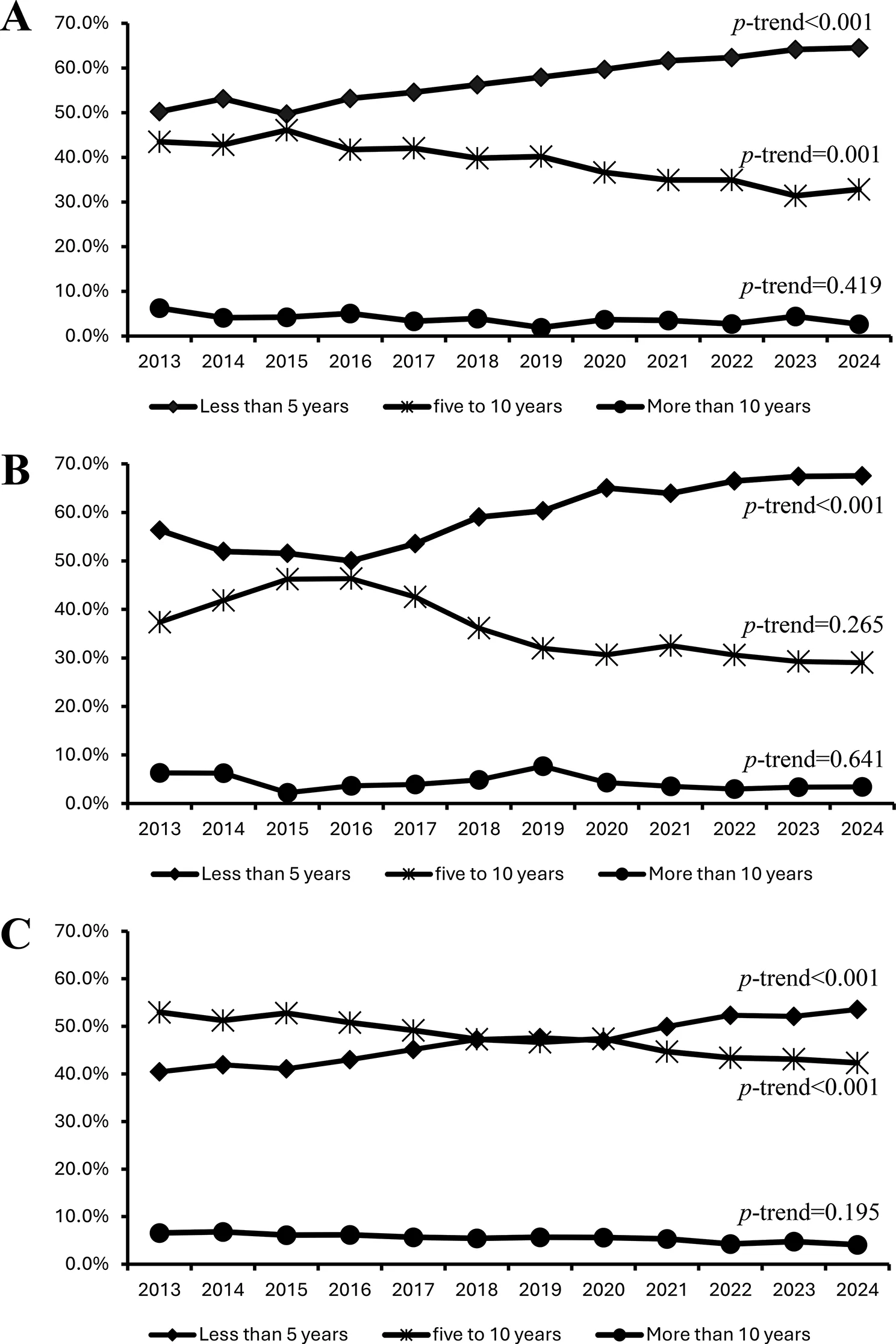
Changes in years intended to practice among late-career General Practitioners in rural/remote (**A**), regional (**B**) and metropolitan (**C**) areas in Australia from 2013 to 2024

Most late-career GPs across all regions were initially registered in Australia or New Zealand, and this proportion increased over time, particularly in rural (2013: 70.4%; 2024: 72.8%) and regional areas (2013: 57.8%; 2024: 68.6%). Consistent with these trends, there was a decline in the relative contribution of overseas-trained GPs in rural (AME: − 0.45; 95% CI: − 0.65 to − 0.24; *p* < 0.001) and regional areas (AME: − 1.45; 95% CI: − 2.00 to − 0.90; *p* < 0.001), while changes in metropolitan areas were small and not statistically significant (AME: − 0.18 pp per year, 95% CI: − 0.43, 0.06, *p* = 0.147). Most late-career GPs had worked for more than 10 years, with this proportion consistently above 97% across rural, regional, and metropolitan areas in 2013 and 2024 (Table 1). There was a small increase in the annual average proportion of GPs with less than ten years of practice in metropolitan areas (AME: 0.06; 95% CI: 0.01, 0.10; *p* = 0.020), no significant change in regional areas (AME: − 0.07; 95% CI: − 0.17, 0.04; *p* = 0.224), and a slight decrease in rural areas (AME: − 0.07; 95% CI: − 0.14, − 0.003; *p* = 0.041) (Table 2). Late-career GPs in rural areas who intend to retire within 5 years increased from 50.2% in 2013 to 65.5% in 2024 (Fig. 2A). This is accompanied by a corresponding decrease in those who intend to continue working for more than 10 years (6.3% in 2013 vs. 2.7% in 2024). A similar pattern of an increase in the proportion of practitioners intending to retire within five years is observed in regional (2013: 56.3%; 2024: 67.5%) and metropolitan areas (2013: 56.3%; 2024: 67.5%). These trends were supported by significant declines in the probability of intending to work less than 5 years compared with longer durations across all regions (all *p* < 0.001) (Table 2).

GPs in rural areas constituted a higher proportion of those working 50 or more hours across all time points (Table 1). There was a shift away from longer working hours across all regions. For instance, relative to those working 1–19 h, there were significant declines in the proportion working 50 h or more (rural: − 4.50 pp per year, 95% CI: − 5.77, − 3.22, *p* < 0.001; regional: − 6.06 pp per year 95% CI: − 8.68, − 3.44, *p* < 0.001; metropolitan: − 4.99 pp per year, 95% CI: − 5.91, − 4.07, *p* < 0.001). Changes in intermediate working hour categories (i.e., 20–34 h and 35–49 h) were more variable across regions (Table 2). Most late-career GPs work in private practice, irrespective of the region (Table 1). However, there was an increase in the proportion of those working in Aboriginal health services (AME: 0.58; 95% CI: 0.48–0.68; *p* < 0.001) and hospitals (AME: 0.39; 95% CI: 0.26–0.51; *p* < 0.001) in rural areas, with smaller but significant changes observed in regional and metropolitan areas (Table 2). Notably, between 95% and 98% of late-career GPs in rural areas worked as clinicians throughout the study period, with the remaining 2–5% in roles such as administration, academia, or research (Supplementary Table 2). Similarly, more than 92% and 96% of practitioners in regional and metropolitan areas worked as clinicians, respectively (Supplementary Table 2).

## Discussion

This study provides the most recent demographic and trend data on the work patterns of late-career GPs in Australia across rural, regional, and metropolitan settings. We observed an increase in the late-career GP workforce across all regions, accompanied by higher female participation and a shift in practice patterns, including moving away from private practice in some regions. However, Indigenous representation remains low. Late-career GPs in rural areas continue to work longer hours than their metropolitan counterparts. The growing intention among these practitioners to retire within the next five years raises significant concerns about workforce shortages, particularly in already underserved rural and remote areas. These findings highlight the critical need for urgent strategies to retain experienced GPs in rural and remote areas and to attract younger practitioners to these regions.

The observed increase in the late-career GP workforce across all geographic regions is consistent with previous reports highlighting the ageing of the Australian medical workforce [2, 15, 16]. The growing dependence on late-career GPs is especially evident in rural and remote areas, where a higher proportion of these practitioners provide services outside private practice, including Aboriginal health services. The relatively higher growth rate of late-career GPs in rural and remote areas may reflect both the ageing of existing practitioners and the slower recruitment of early-career GPs into these regions [1, 2, 5]. This pattern raises concerns about future workforce sustainability, particularly as older GPs approach retirement and demand for primary care services in these areas remains high [5, 16]. Recent evidence demonstrates that workforce challenges in rural health are multifaceted and encompass more than shortages alone [17, 18]. Describing the rural health workforce as merely a shortage issue neglects the social, professional, and community dynamics that influence recruitment and retention [19]. For example, factors such as sense of place, belonging, and place attachment play a significant role in practitioners’ decisions to remain in or leave rural areas [20]. Hence, the increasing dependence on late-career GPs in rural areas observed in our study may reflect not only difficulties recruiting younger practitioners but also broader structural factors affecting workforce retention and service delivery in rural areas [18]. The small number of Indigenous late-career GPs reflects longstanding concerns about Indigenous participation in the medical profession [21, 22]. Initiatives such as those led by Medical Deans Australia and New Zealand and the Australian Indigenous Doctors’ Association (AIDA) have contributed to increased admission of Indigenous medical students [23–25], but sustained efforts are required to support career progression into later stages of practice. Late-career Indigenous practitioners play a critical role in delivering culturally safe care, improving health outcomes for Aboriginal and Torres Strait Islander communities, and serving as role models for future generations [26].

The increasing proportion of female late-career GPs across all regions is encouraging, as it suggests improved retention and career longevity for women in general practice [27, 28]. However, the late-career GP workforce remains male-dominated, particularly in rural and regional areas [8]. Gender disparity in the rural and regional GP workforce, particularly among more experienced practitioners, may have implications for access to care. For example, women in rural and remote areas are less likely to visit GPs frequently compared to their counterparts in metropolitan areas [29]. While factors including socioeconomic status and transportation difficulties have been suggested as possible causes of these inequalities, the availability of female GPs has also been implicated [29]. For some women, access to a female GP is not merely a preference but influences willingness to seek care, particularly for sensitive or preventive health services [30]. Policy efforts to improve gender diversity in the Australian medical workforce, including increased admissions of female medical students and greater participation in GP training, are likely contributing to these observed trends [27, 28, 31]. Nevertheless, achieving workforce equity may require consideration of differences in working patterns, as female practitioners have historically worked fewer hours on average [32, 33].

The growing proportion of locally trained late-career GPs, alongside a relative decline in overseas-trained practitioners in rural and regional areas, likely reflects increased domestic training capacity, improved retention of locally trained doctors, and changing migration dynamics [27, 28, 31, 34]. Importantly, this proportional decline occurred alongside continued growth in the absolute number of overseas-trained practitioners, indicating that the observed pattern reflects differential growth rather than a contraction in international supply. The increasing expansion of Australian medical education and GP training pathways over time potentially contributed to a cohort replacement effect, whereby locally trained practitioners comprise an increasing share of the workforce [27, 28, 31]. In addition, overseas-trained doctors recruited in earlier decades are now reaching retirement age, which may further influence these trends. Regulatory policies such as Section 19AB, which require overseas-trained doctors to work for 10 years in rural priority areas, may also shape long-term workforce distribution and retention patterns [34]. While increasing domestic capacity is encouraging for workforce sustainability [8]. Nevertheless, continued monitoring is essential to ensure that reduced reliance on overseas-trained doctors does not adversely affect service delivery in rural and remote areas.

The observed trend toward reduced working hours and increasing intention to retire within the next five years is consistent with reports from other high-income countries [35–38]. Notably, the increasing intention to retire within five years was more pronounced among late career GPs between the 65- and 74-year-old category and not among those aged 75 years and older (Supplementary Fig. 1), suggesting that the patterns observed in our study may be driven by the working preference of the 65–74 age group. These findings raise significant concerns about the future availability of experienced practitioners, especially in rural communities where workforce supply is already constrained. The decision of GPs to retire early or continue working in underserved or rural areas is shaped by complex push and pull factors, including career stage, professional support, community integration, and opportunities for meaningful work [19, 39]. For example, in the United Kingdom, pre-retirement migration of older GPs to rural areas is linked to lifestyle preferences, community engagement, and the prospect of a gradual transition toward retirement [40]. These findings highlight the need for GP retention strategies that move beyond simply addressing professional incentives to creating environments where GPs feel a sense of belonging, professional fulfilment, and community connection [20, 40]. Burnout, administrative burden, and evolving work–life priorities have also been identified as key drivers of reduced work hours and retirement intentions among GPs [5, 16]. Addressing these workforce challenges will be critical to ensuring ongoing access to high-quality primary care, particularly in rural and remote areas. Policies that support flexible transition to retirement, promote workload redistribution, and facilitate intergenerational knowledge transfer are urgently needed to preserve the expertise of late-career GPs [5].

### Limitations

This study has several limitations. First, the analysis relied on self-reported intentions regarding future work and retirement, which may be subject to response bias [41]. However, prior studies have demonstrated a reasonable correlation between stated intentions and subsequent retirement among doctors [38], suggesting that our findings are likely to reflect true trends. Also, we are unable to capture participants who work across metropolitan, regional, rural and remote areas. This is because the survey requires participants to provide only the location of their principal place of practice. Furthermore, the study did not examine qualitative aspects of late-career practice, such as motivations for continued work or reasons for relocation between geographic regions. Recent Australian and international qualitative studies suggest that these factors, including organisational support, professional identity, place attachment, community belonging, family considerations and lifestyle preferences, may substantially influence recruitment and retention decisions among rural health professionals [17, 20, 42]. Similarly, emerging evidence suggests that some doctors intentionally relocate to rural areas later in their careers for lifestyle and professional reasons [40]. Nonetheless, the large sample size and nationally representative dataset used in this study provide a robust basis for understanding overarching patterns for workforce planning.

In conclusion, the late-career GP workforce in Australia is growing, becoming more gender-diverse, and increasingly locally trained, but continues to face challenges with Indigenous representation and impending retirements. Addressing rural GP workforce sustainability may require more than increasing training numbers. Strategies to improve retention, professional support, flexible career pathways, and community integration may be warranted for maintaining access to primary care services in rural and remote communities.

## Supplementary Information

Below is the link to the electronic supplementary material.

Supplementary Material 1

## Data Availability

The data supporting the findings of this study are available from the Health Workforce Data Tool, managed by the Australian Health Practitioner Regulation Agency (AHPRA), in conjunction with the National Boards and the Australian Government Department of Health and Aged Care. The Health Workforce Data Tool is available to the general public.

## Abbreviations

AHPRA: Australian Health Practitioner Regulation Agency
AIDA: Australian Indigenous Doctors’ Association
AIHW: Australian Institute of Health and Welfare
AME: Average Marginal Effects
GPs: General Practitioners
MMM: Modified Monash Model
NHWDS: National Health Workforce Dataset
RHMT: Rural Health Multidisciplinary Training
