# Supplementary material for: How have the demographic and practice patterns of older general practitioners in rural and remote Australia changed over the past decade?
Source: BMC Health Serv Res. 2026 Jul 30;26:1491. doi: 10.1186/s12913-026-15090-0 (PMC13726073; doi:10.1186/s12913-026-15090-0)
Supplement: Supplementary file 1 [file 12913_2026_15090_MOESM1_ESM.docx]

Supplementary Tables and Figure

| **A** |  |
| --- | --- |
| **B** |  |
| Supplementary Figure 1: Changes in years intending to retire among late-career general practitioners in the 65–74 age group (A), 75 years and older age category (B) from 2013 to 2024. | |

|  | Less than 5 years | five to 10 years | More than 10 years |
| --- | --- | --- | --- |
| 2013 | 49.5327% | 44.1121% | 6.35514% |
| 2014 | 49.5652% | 45.3913% | 5.04348% |
| 2015 | 47.2699% | 48.6739% | 4.05616% |
| 2016 | 49.564% | 45.6395% | 4.79651% |
| 2017 | 52.2088% | 44.3106% | 3.48059% |
| 2018 | 55.0562% | 40.4494% | 4.49438% |
| 2019 | 56.3446% | 39.9302% | 3.72526% |
| 2020 | 60.2754% | 35.6992% | 4.02542% |
| 2021 | 61.0656% | 35.3484% | 3.58607% |
| 2022 | 63.2219% | 33.9412% | 2.83688% |
| 2023 | 65.1187% | 30.6502% | 4.23117% |
| 2024 | 64.503% | 32.6572% | 2.83976% |

|  | Less than 5 years | five to 10 years | More than 10 years |
| --- | --- | --- | --- |
| 2013 | 74.359% | 17.9487% | 7.69231% |
| 2014 | 74.0741% | 20.3704% | 5.55556% |
| 2015 | 70.9677% | 29.0323% | 0% |
| 2016 | 75% | 20.5882% | 4.41176% |
| 2017 | 66.6667% | 27.5362% | 5.7971% |
| 2018 | 67.4699% | 28.9157% | 3.61446% |
| 2019 | 68.1818% | 27.2727% | 4.54545% |
| 2020 | 67.6768% | 32.3232% | 0% |
| 2021 | 69.3878% | 26.5306% | 4.08163% |
| 2022 | 60.9091% | 35.4545% | 3.63636% |
| 2023 | 61.6541% | 33.8346% | 4.51128% |
| 2024 | 67.8322% | 28.6713% | 3.4965% |

….

| **Supplementary Table 1: Number of General Practitioners aged 65 years and older stratified by MMM classification in 2019 and 2023** | | | | | | | | | | | | | |
| --- | --- | --- | --- | --- | --- | --- | --- | --- | --- | --- | --- | --- | --- |
|  | **Year** | **2013** | **2014** | **2015** | **2016** | **2017** | **2018** | **2019** | **2020** | **2021** | **2022** | **2023** | **2024** |
| Metropolitan | 2019 | 2523 | 2726 | 2949 | 3142 | 3323 | 3454 | 3613 | 3696 | 4065 | 4086 | 4097 | 4226 |
|  | 2023 | 2520 | 2724 | 2946 | 3141 | 3320 | 3449 | 3611 | 3691 | 4060 | 4081 | 4094 | 4225 |
| Regional | 2019 | 209 | 213 | 230 | 248 | 258 | 289 | 303 | 302 | 340 | 341 | 330 | 350 |
|  | 2023 | 212 | 215 | 233 | 249 | 261 | 294 | 304 | 307 | 345 | 346 | 333 | 351 |
| Rural | 2019 | 454 | 493 | 546 | 568 | 612 | 645 | 696 | 795 | 796 | 824 | 825 | 829 |
|  | 2023 | 454 | 493 | 546 | 568 | 612 | 645 | 697 | 795 | 796 | 824 | 825 | 829 |

…

| **Supplementary Table 2: Job roles of the study participants stratified by the location of practice** | | | | | | | | | | | | | |
| --- | --- | --- | --- | --- | --- | --- | --- | --- | --- | --- | --- | --- | --- |
| Regions | Job Roles | 2013 | 2014 | 2015 | 2016 | 2017 | 2018 | 2019 | 2020 | 2021 | 2022 | 2023 | 2024 |
| Metropolitan | Clinicians | 2448 (97.0%) | 2661 (97.6%) | 2856 (96.8%) | 3029 (96.4%) | 3215 (96.7%) | 3323 (96.2%) | 3478 (96.3%) | 3571 (96.6%) | 3920 (96.4%) | 3929 (96.2%) | 3955 (96.5%) | 4071 (96.3%) |
|  | Others* | 75 (3.0%) | 65 (2.4%) | 93 (3.2%) | 113 (3.6%) | 108 (3.3%) | 131 (3.8%) | 135 (3.7%) | 125 (3.4%) | 145 (3.6%) | 157 (3.8%) | 142 (3.5%) | 155 (3.7%) |
| Regional | Clinicians | 207 (98.6%) | 203 (95.3%) | 223 (97.0%) | 241 (97.2%) | 245 (95.0%) | 277 (95.8%) | 285 (94.1%) | 288 (95.4%) | 319 (93.8%) | 321 (94.1%) | 306 (92.7%) | 331 (94.6%) |
|  | Others* | 3 (1.4%) | 10 (4.7%) | 7 (3.0%) | 7 (2.8%) | 13 (5.0%) | 12 (4.2%) | 18 (5.9%) | 14 (4.6%) | 21 (6.2%) | 20 (5.9%) | 24 (7.3%) | 19 (5.4%) |
| Rural | Clinicians | 445 (98.0%) | 473 (95.9%) | 524 (96.0%) | 544 (95.8%) | 587 (95.9%) | 618 (95.8%) | 669 (96.1%) | 757 (95.2%) | 761 (95.6%) | 802 (97.3%) | 797 (96.6%) | 799 (96.4%) |
|  | Others* | 9 (2.0%) | 20 (4.1%) | 22 (4.0%) | 24 (4.2%) | 25 (4.1%) | 27 (4.2%) | 27 (3.9%) | 38 (4.8%) | 35 (4.4%) | 22 (2.7%) | 28 (3.4%) | 30 (3.6%) |
| *Others include administrators, academics and researchers | | | | | | | | | | | | | |
